# Impact of Tumor Localization on Early Recurrence After Curative Resection in Pancreatic Ductal Adenocarcinoma

**DOI:** 10.3390/medicina61101799

**Published:** 2025-10-06

**Authors:** Eda Caliskan Yildirim, Ilkay Tugba Unek, Ilhan Oztop, Mehmet Uzun, Tarkan Unek, Ozgul Sagol

**Affiliations:** 1 Division of Medical Oncology, Department of Internal Medicine, Dokuz Eylul University, Izmir 35340, Türkiye; memed.uzun3846@gmail.com; 2 Division of Medical Oncology, Institute of Oncology, Dokuz Eylul University, Izmir 35340, Türkiye; ilkaytugbaunek@gmail.com (I.T.U.); ilhan.oztop@deu.edu.tr (I.O.); ozgul.sagol@deu.edu.tr (O.S.); 3 Department of General Surgery, Dokuz Eylul University, Izmir 35340, Türkiye; tarkan.unek@deu.edu.tr; 4 Department of Pathology, Dokuz Eylul University, Izmir 35340, Türkiye

**Keywords:** pancreatic ductal adenocarcinoma, early recurrence, tumor localization, CA 19-9, neoadjuvant therapy, resectable PDAC

## Abstract

*Background and objectives*: Early recurrence (ER) following curative-intent surgery for pancreatic ductal adenocarcinoma (PDAC) is associated with poor prognosis. Identifying preoperative risk factors for ER is essential for optimizing perioperative strategies. This study aimed to investigate perioperative predictors of ER, with a specific focus on tumor localization. *Methods*: We retrospectively analyzed 163 patients who underwent R0 or R1 resection for PDAC. ER was defined as recurrence within 6 months postoperatively. Two separate multivariate logistic regression analyses were conducted: one including only preoperative variables, and one including both pre- and postoperative factors. *Results*: ER occurred in 35.6% of patients and was associated with significantly worse overall survival (median 9 vs. 21 months, *p* < 0.001) and post-recurrence survival (5 vs. 8 months, *p* = 0.008). Preoperative ECOG performance status > 0 (OR 3.31, *p* = 0.013) and CA 19-9 > 208 U/mL (OR 3.18, *p* = 0.022) were identified as independent predictors of ER. In the postoperative model, tumor localization in the body/tail (OR 3.23, *p* = 0.035), tumor size > 3.25 cm, lymph node ratio > 0.13, and absence of adjuvant therapy were also significant. Notably, tumor location did not influence overall survival. *Conclusions*: Tumor localization in the body/tail of the pancreas is independently associated with early recurrence but not overall survival. These findings highlight the importance of incorporating tumor site into preoperative risk stratification and support the consideration of neoadjuvant therapy in select anatomically resectable patients, particularly those with left-sided tumors.

## 1. Introduction

Pancreatic ductal adenocarcinoma (PDAC) accounts for more than 90% of all pancreatic malignancies and remains one of the most lethal solid tumors. It is currently the sixth leading cause of cancer-related death worldwide [1]. Although radical resection is the only curative treatment option for PDAC patients, only 15–20% of patients are eligible for surgery at the time of diagnosis [2]. Despite optimal adjuvant treatment approaches, 80% of patients relapse within a short time. Recurrence within the first 12 months is observed in more than 50% of patients who have undergone surgery for PDAC [3].

Identifying patients at high risk for early recurrence (ER) is crucial for optimizing treatment strategies and avoiding unnecessary surgical morbidity. ER is generally defined as recurrence within 6 to 12 months following surgery and is associated with extremely poor survival outcomes [4].

Several perioperative factors have been linked to ER, including elevated carbohydrate antigen 19-9 (CA 19-9) levels, large tumor size, high metastatic lymph node ratio (mLNR), and poor tumor differentiation [5,6,7,8,9]. However, the prognostic value of tumor localization within the pancreas (head/neck vs. body/tail) remains unclear and under-investigated.

Tumor location may influence biological behavior, surgical approach, lymphatic drainage, and gene expression profiles in PDAC. While some studies have suggested better outcomes in body/tail tumors [10], others report a more aggressive phenotype and higher proliferative potential. The International Pancreatic Cancer Genome Consortium, for instance, has proposed that tumors arising from the body and tail demonstrate distinct molecular features—including greater epithelial–mesenchymal transition activity—compared to tumors of the pancreatic head [11]. Despite this, few studies have evaluated the relationship between tumor localization and early recurrence following curative resection.

In the current study, we sought to identify perioperative clinicopathological factors associated with early recurrence after curative surgery for PDAC, with a specific focus on the impact of tumor localization. We hypothesized that tumors located in the body and tail of the pancreas are more likely to relapse early. We aimed to determine whether localization serves as an independent predictor of ER. These findings could help refine patient selection for neoadjuvant therapy, even in anatomically resectable cases, and may contribute to individualized treatment planning in PDAC.

## 2. Methods

### 2.1. Study Population

Between January 2000–June 2020, 196 patients who underwent curative intent resection for PDAC at Dokuz Eylul University Hospital, Izmir, Turkey, were included in this study. All patients were histologically confirmed by a pathologist specializing in the pancreaticobiliary system. Patients who had intraductal papillary mucinous carcinoma (n = 5), R2 resection (n = 2), <6 months follow-up (n = 12), and insufficient information on recurrence (n = 14) were excluded. After excluding 33 patients, data from the remaining 163 patients were retrospectively analyzed. Tumours were classified as head/neck (H/N) versus body/tail (B/T) based on their dominant anatomical epicentre on pre-operative imaging and confirmed on pathology. The Local Ethics Committee approved this study.

If there was no contraindication for contrast agent application, all patients underwent preoperative staging with contrast-enhanced multidetector CT. From 2010 onwards, MRI was increasingly used to assess biliary and vascular involvement, and PET-CT was occasionally applied in cases with equivocal findings. Staging was assigned according to the AJCC 8th edition criteria.

The resectability decision was made by the pancreaticobiliary multidisciplinary team based on the tumor’s contact with adjacent arteries and veins.

Data regarding preoperative and postoperative demographics, clinicopathologic features, and treatment details were extracted from the institutional database. Preoperative CA19-9 levels were recorded. The type of surgical procedure was classified as Whipple operation or distal pancreatectomy + splenectomy with regional lymph node dissection. The mLNR was calculated by dividing the metastatic lymph node count by the resected lymph node count. The pathologic stage of the patients was updated according to the new staging system (American Joint Committee on Cancer [AJCC] Tumor, Node, Metastasis [TNM] Staging of Pancreatic Cancer, 8th ed., 2017) by a pathologist because the TNM staging for pancreatic cancer changed in the surveillance period of the study. Margin status was evaluated according to the College of American Pathologists (CAP) guidelines, defining R1 as tumour cells within 1 mm of the resection margin. Patients with a previous history of undergoing chemotherapy and radiotherapy were excluded from the study.

### 2.2. Adjuvant Therapy

Postoperative 6 months of systemic adjuvant chemotherapy was planned. Adjuvant chemotherapy was initiated within 3 months postoperatively for all patients who were considered eligible for chemotherapy. Gemcitabine-based chemotherapy was the predominant regimen. During the earlier decade (2000–2009), adjuvant treatment most commonly consisted of gemcitabine monotherapy and/or 5-FU with folinic acid. In the later decade (2010–2020), gemcitabine-capecitabine (GemCap) became the preferred regimen, and FOLFIRINOX was increasingly administered to younger and fit patients. In 52% of cases, radiotherapy (45 Gy in 1.8 Gy fractions) was administered sequentially after completion of chemotherapy. Importantly, no patients received radiotherapy alone. Given the retrospective design and heterogeneity across two decades, adjuvant treatment was incorporated into the postoperative model as a binary covariate (presence vs. absence of chemotherapy ± radiotherapy).

### 2.3. Follow-Up and Recurrence

Patients were followed up by a medical oncologist at the outpatient clinic at our hospital every 3 months within the first 2 years after surgery. The follow-up examinations included a hemogram, liver and renal function tests, CA19-9 levels, and enhanced CT of the thorax, abdomen, and pelvis. If there was a suspicion of recurrence with these examinations, MRI, and/or 18F-fluorodeoxyglucose positron emission tomography/CT (PET-CT) were added to the evaluation.

After the imaging methods detected the recurrence, the recurrence region was grouped as local, liver, lung, peritoneum, other, or multiple-detection simultaneously in more than one region. Early recurrence (ER) was defined as a recurrence within the first 6 months after surgery, while the recurrence of more than 6 months and non-recurrent disease after surgery was described as non-early recurrence (non-ER).

### 2.4. Statistical Analysis

The demographic and clinicopathological features of the patients were compared between the head and neck group and the body and tail group. The categorical variables were compared using the χ^2^ test; the continuous variables were compared using a Student’s *t*-test or the Mann–Whitney U test. The receiver operating characteristic (ROC) curve was used to determine the optimal cutoff values of the continuous variables (pathological tumor size, CA19-9 levels, and LNR) for ER prediction. To explore potential temporal effects, patients were also stratified into two treatment periods (2000–2009 vs. 2010–2020), and early recurrence rates were compared between these eras. Two separate multivariate logistic regression analyses (one for preoperative and one for postoperative) were used to assess the potential independent risk factors associated with ER. To assess collinearity, we generated a correlation matrix and calculated variance inflation factors (VIFs) for all independent variables. VIF values around 1.1 indicated that multicollinearity was not a significant concern. Sensitivity analyses were additionally performed using alternative definitions of early recurrence at 3 and 12 months, in addition to the main 6-month threshold. Survival was calculated using the Kaplan–Meier method and was compared between the groups using the log-rank test.

All statistical analyses were performed with the SPSS Statistics 25.0 for iOS software program (SPSS, Inc., Chicago, IL, USA), and *p* < 0.05 was considered statistically significant.

## 3. Results

Of the patients who underwent curative resection for PDCA, 56% (n = 92) were male and 44% (n = 71) were female. The mean age ±standard deviation (SD) was 61 ± 9 years. It was observed that the tumor was located in the pancreatic head in 77.3% (n = 126) of the patients, while in the pancreas body and tail in 22.7% (n = 37) of the patients. An R0 resection was done in 53.4% (n = 87) of the patients; the rest of the cohort underwent an R1 resection. Most of the patients had T3-T4 tumors (74.2%) or nod-positive (77.3%) disease. Preoperative serum CA 19-9 levels were observed as a median of 294 U/mL (IQR: 64–954). The demographic and clinicopathologic characteristics of the entire study population and subgroups are summarized in Table 1. Adjuvant treatment was given to 80.4% of the patients following curative resection., while 19.6% of the patients could not receive any adjuvant treatment mainly due to poor postoperative performance status or surgical complications. Among those treated, 31% received chemotherapy alone, and 52.3% received sequentially chemotherapy followed by radiotherapy. Gemcitabine-based chemotherapy was administered to 92.3% of the patients. During the follow-up period, 85.8% (n = 140) of the patients relapsed, and 14.1% (n = 23) did not relapse. ER occurred in 35.6% overall, affecting 30.4% (38/126) of H/N tumors versus 54.1% (20/37) of B/T tumors (*p* = 0.008). When stratified by treatment period, the incidence of early recurrence within 6 months was similar between patients operated on in 2000–2009 and those in 2010–2020 (34.7% vs. 36.8%, *p* = 0.87).

ROC analysis identified cut-offs of 3.25 cm for tumour size (AUC = 0.657; *p* = 0.017), 208 U/mL for CA19-9 (AUC = 0.651; *p* = 0.022), and 0.13 for LNR (AUC = 0.690; *p* = 0.004).

An analysis of the recurrence patterns of the patients showed that 67.2% of the recurrences in the ER group occurred simultaneously in more than one metastasis region. In contrast, in the non-ER group, this rate was 46.3% (*p* = 0.014) (Table 2). No significant differences in recurrence patterns were observed between tumors located in the head/neck and those in the body/tail of the pancreas.

The median follow-up of the study population was 116 months (IQR: 79-152). While 11 patients with no recurrence were continuing their disease-free follow-up, 12 patients died due to non-cancer reasons. The median time to recurrence in the entire cohort was 7 months (IQR: 5–15). The median disease-free survival (DFS) of patients with ER and non-ER was 4 months (IQR: 2–5) and 12 months (IQR: 9–20), respectively. When stratified by location, median DFS was 10 months for H/N versus 6 months for B/T tumors (log-rank *p* = 0.019).

**Figure 1:**
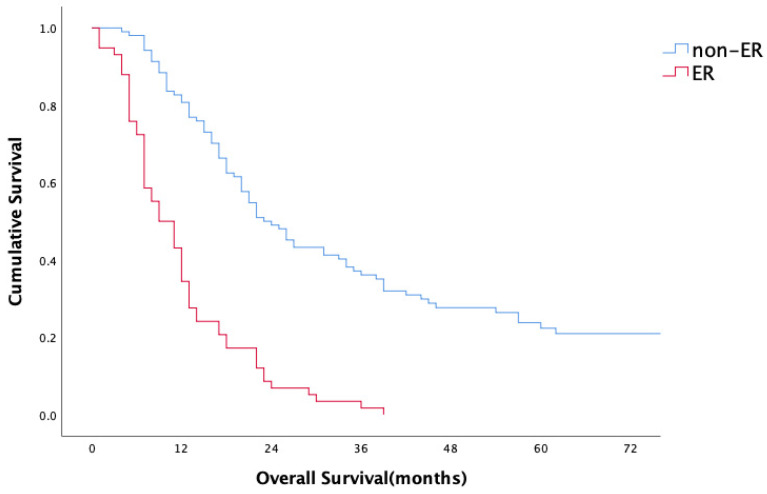
Overall survival.

**Figure 2:**
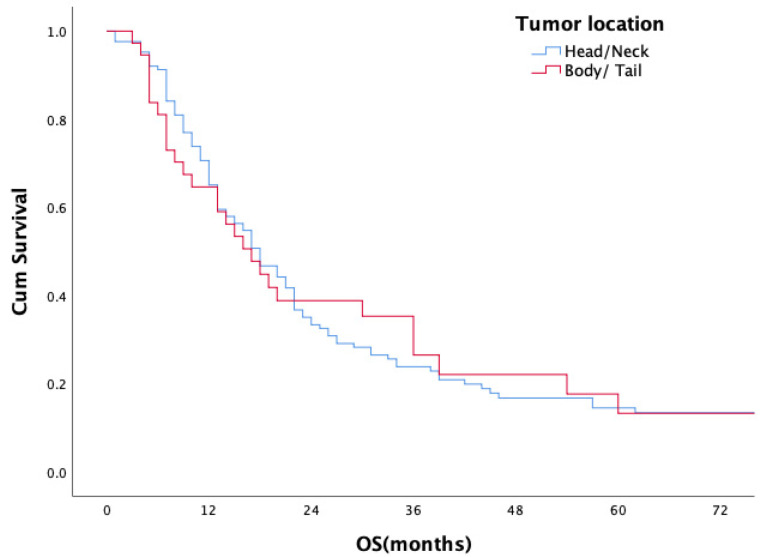
Overall survival according to tumor location.

**Figure 3:**
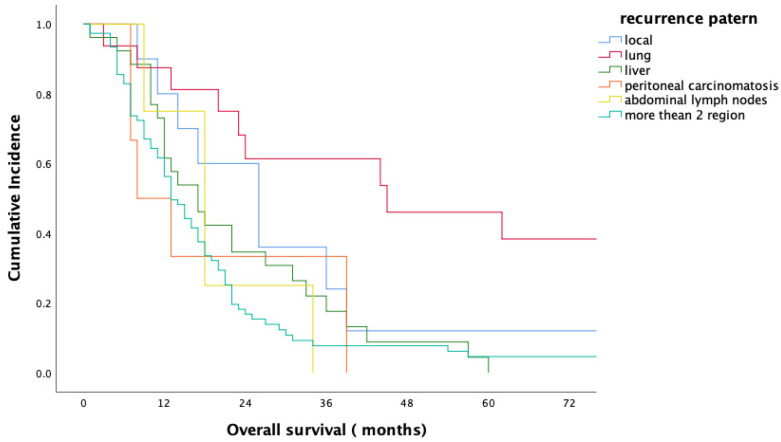
Overall survival according to the metastasis region.

For the entire study population, the median overall survival (OS) period was 18 months (range 1–165 months); the 1- and 2-year survival rates were 45.9% and 28.7%, respectively. Median OS, 1-year and 2-year OS rates of the patients with ER were 9 months, 43% and 8%, respectively, in comparison to 21 months, 82% and 47% for patients in the non-ER group (*p* < 0.001) (Figure 1). No statistically significant difference in overall survival was observed between head/neck and body/tail tumors (18 m vs. 17 m, *p* = 0.893, Figure 2). Evaluation of the OS according to metastasis region showed that all relapsed patients with isolated lung metastases had statistically significantly longer OS in comparison to the patients with other sites of metastasis (median 44 months, *p* = 0.001) (Figure 3).

### Perioperative Risk Factor Analysis

The factors predicting ER were analyzed in both groups, preoperatively and postoperatively, and two separate logistic regression analyses were performed. Among the preoperative risk factors, Eastern Cooperative Oncology Group (ECOG) performance status > 0 (OR: 3.31, 95% CI: 1.28–8.55, *p* = 0.013) and CA 19-9 level > 208 U/mL (OR: 3.18, 95% CI: 1.18–8.57, *p* = 0.022) were identified as independent risk factors for ER (Table 3). Among the postoperative risk factors, four were found to be correlated with ER: tumor localization (OR: 3.23 for body-tail location, 95% CI: 1.08–9.64, *p* = 0.035), pathological tumor size > 3.25 cm (OR: 3.32, 95% CI: 1.21–9.04, *p* = 0.019), LNR > 0.13 (OR: 3.49, 95% CI: 1.34–9.08, *p* = 0.01), and no adjuvant chemotherapy (OR: 6.63, 95% CI: 1.81–24.72, *p* = 0.004) (Table 4). Tumor localisation did not reach significance in the preoperative model (OR 2.55, *p* = 0.076) but was significant in the postoperative model (OR 3.23, *p* = 0.035).

In sensitivity analyses, tumour localisation (body/tail vs. head/neck) remained a significant predictor of recurrence at 3 and 6 months, but lost statistical significance when recurrence was defined within 12 months (Supplementary Table S1).

## 4. Discussion

In this study, more than 80% of PDAC patients who underwent curative-intent surgery experienced recurrence, a figure consistent with previous literature. Early recurrence (ER), defined as relapse within six months of surgery, occurred in 35.6% of patients and was associated with significantly worse outcomes. Median overall survival (OS) in the ER group was 9 months, compared to 21 months in the non-ER group. Patients with ER were more likely to present with multiple-site metastases at the time of recurrence (*p* = 0.014). Notably, patients with isolated lung metastasis had significantly longer survival (median 44 months, *p* = 0.001), suggesting a distinct biologic behavior. Overall, nearly one-third of patients recurred during or shortly after adjuvant therapy, highlighting the importance of accurate preoperative risk stratification.

Compared to prior reports, our observed ER rate of 35.6% is relatively high. Most studies report ER in 20–25% of resected PDAC patients within 6 months5, 6, 9, although studies including borderline resectable (BR) cases or omitting neoadjuvant therapy often report rates closer to 40%9. In our cohort, the high ER rate likely reflects the inclusion of more stage III tumors and the use of upfront surgery without neoadjuvant treatment in anatomically resectable but biologically aggressive tumors. While the OS of ER patients was in line with existing studies (8–11 months), the non-ER group in our study had slightly shorter OS, likely due to higher baseline disease burden. The effect of treatment era was also explored. While early recurrence rates were comparable between 2000–2009 and 2010–2020 (34.7% vs. 36.8%), overall survival improved in the later decade, reflecting advances in systemic therapy options and supportive care. This indicates that temporal changes did not affect the risk of early recurrence, but survival outcomes benefited from evolving management strategies.

To distinguish between preoperative and postoperative predictors of early recurrence, we performed two separate multivariate logistic regression models. This dual-model strategy enabled us to assess the impact of both early, clinically available markers and final histopathological features in predicting biologically aggressive disease. Importantly, tumor localization was identified as an independent predictor of early recurrence—a novel finding in the perioperative setting. Body/tail tumor location was the sole independent preoperative predictor of ultra-early (≤3 months) recurrence in a recent study [12]. Although the prognostic role of tumor location in PDAC has been debated, most studies have focused on overall survival or stage at diagnosis, with conflicting results. While some SEER-based analyses report improved survival in body/tail tumors [13], others demonstrate more aggressive molecular phenotypes in these tumors, including higher proliferative indices and increased epithelial–mesenchymal transition (EMT) activity [14]. The Australian Pancreatic Cancer Genome Initiative, for example, highlighted biologically distinct gene expression patterns in body/tail tumors compared to those in the head/neck [11]. Although our study did not include molecular or transcriptomic analyses, our findings are consistent with these previous reports. They may reflect an underlying biological aggressiveness that manifests early, even in patients undergoing upfront surgery.

Interestingly, while tumor localization is a known preoperative variable, it did not reach statistical significance in the preoperative multivariate model. We explored potential collinearity by performing correlation and collinearity diagnostics: the correlation matrix showed only weak-to-moderate associations, and variance inflation factor (VIF) values were ~1.1 for all variables, indicating no significant multicollinearity (Supplementary Table S2). Therefore, the lack of significance in the preoperative model may instead reflect the limited discriminative power of preoperative variables compared with postoperative pathological data. The independent prognostic effect of body/tail location became more evident in the postoperative model, likely because it better captures the downstream pathological consequences of aggressive tumor biology. Nevertheless, the modest sample size may have introduced some model instability, which should be considered when interpreting these findings.

Despite its association with early recurrence, tumor localization did not influence overall survival in our cohort. This may be explained by the survival-extending effects of adjuvant and salvage therapies administered after recurrence, or by the limited sample size in the body/tail subgroup. The absence of an overall survival difference despite earlier recurrence in body/tail tumors underscores the importance of using tumor location as a preoperative risk marker, rather than a stand-alone prognostic factor.

Our sensitivity analyses further demonstrated that tumour localisation predicted recurrence when defined at 3 and 6 months, but not at 12 months. This finding suggests that localisation is primarily a marker of biologically aggressive behaviour leading to ultra-early relapse, whereas its prognostic effect becomes diluted over longer timeframes as other risk factors dominate. These results reinforce the concept that localisation should be particularly considered when identifying patients at risk of early recurrence who may benefit from neoadjuvant approaches. Several randomized trials investigating the role of neoadjuvant therapy in resectable PDAC have failed to demonstrate a survival benefit in the overall population [15]. However, in these studies, patients with body or tail tumors represented only approximately 25% of enrolled cases, potentially underpowering subgroup analyses [16,17]. Recently, a large international multicenter study evaluated resectable left-sided pancreatic cancers and showed that neoadjuvant therapy significantly improved overall survival in this subgroup [18]. These findings, in line with our data, suggest that body/tail tumors may require distinct treatment algorithms, and tumor location should be actively considered when tailoring preoperative strategies, even in anatomically resectable patients.

Our study also reinforces the value of CA 19-9 and ECOG performance status as preoperative biomarkers. While current guidelines suggest CA 19-9 > 500 U/mL as a potential sign of biologically borderline resectability [19], our study and others report lower thresholds (e.g., 133–208 U/mL) associated with early relapse [5,6,20]. Similarly, even a modest reduction in performance status (ECOG 1–2) was associated with worse outcomes [21]. The role of these variables in refining treatment strategy—especially when combined with tumor location—warrants further prospective validation.

This study has several limitations. It is a single-center retrospective analysis with a modest sample size, particularly in the body/tail subgroup. Some patients with borderline resectable disease underwent upfront surgery, possibly enriching the cohort for biologically aggressive tumors. Our relatively high R1 rate (47%) reflects the use of the CAP definition (≤1 mm rule), which yields higher R1 rates compared to the 0 mm rule. Moreover, the inclusion of patients with advanced T3–T4 disease and some borderline resectable tumours in our cohort likely contributed further to this rate. The tumour size cut-off (3.25 cm) was determined using pathological measurements, as preoperative imaging data were not consistently available in our retrospective cohort. This limits its utility as a purely preoperative marker and should be considered when interpreting the results. We acknowledge that ROC AUCs were modest; therefore, cut-offs were used to aid risk stratification within multivariable models rather than as definitive diagnostic thresholds (Supplementary Table S3). Another limitation relates to the heterogeneity of adjuvant treatment. Radiotherapy was only delivered sequentially after chemotherapy, and no patients received radiotherapy alone. As our study was not designed to evaluate local control, we analysed adjuvant therapy simply as a binary covariate (chemotherapy ± radiotherapy vs. none). In this framework, the absence of adjuvant therapy remained an independent predictor of early recurrence. We did not assess Lewis antigen status, which could affect CA 19-9 interpretation, and genomic profiling was not available.

## 5. Conclusions

Our findings suggest that tumor location in the body/tail of the pancreas is an independent predictor of early recurrence following curative-intent surgery for PDAC. While it did not independently influence overall survival, it may reflect biologic aggressiveness not captured by anatomical resectability alone. Combined with CA 19-9 and performance status, tumor localization may serve as a valuable preoperative marker to guide neoadjuvant treatment decisions and improve long-term outcomes.

## Figures and Tables

**Table 1:** Demographic and clinicopathologic characteristics of the study population.

	All Patients (n = 163)	Head-Neck (n = 126)	Body-Tail (n = 37)	*p*-Value
Male, n (%)	92 (56.4)	72 (57.1)	20 (54.1)	0.73
Age, years, mean (SD) <70 years old >70 years old	61 (9) 137 (84) 26 (16)	61 (9) 107 (84.9) 19 (15.1)	60 (8) 30 (81.1) 7 (18.9)	0.57
ECOG, n (%) 0 1–2	101 (62.7) 60 (37.3)	77 (62.1) 47 (37.9)	24 (64.9) 13 (35.1)	0.76
Charlson comorbidity index, median (IQR)	2 (1–4)	2 (1–4)	2 (1–4)	0.92
Preoperative CA19-9 level (U/mL), median (IQR)	294 (64–954)	225 (64–892)	464 (59–2021)	0.46
Pathological tumor size, mean (SD)	3.5 (1.5)	3.5 (1.2)	4.5 (1.8)	0.03
T stage, n (%) T1–T2 T3–T4	42 (25.8) 121 (74.2)	32 (25.4) 94 (74.6)	10 (27) 27 (73)	0.83
Positive lymph nodes, n (%)	126 (77.3)	95 (75.4)	31 (83.8)	0.37
TNM stage, n (%) Stage 1–2 Stage 3–4	103 (63.1) 60 (26.9)	82 (65.1) 44 (34.9)	21 (56.8) 16 (43.2)	0.43
Residual tumor, n (%) R0 R1	87 (53.4) 76 (46.6)	61 (48.4) 65 (51.6)	26 (70.3) 11 (29.7)	0.02
Differentiation, n (%) well modified-poor	84 (54.5) 70 (45.5)	62 (51.2) 59 ((48.8)	22 (66.7) 11 (33.3)	0.16
Lymphovascular invasion, n (%)	127 (80.4)	98 (80.3)	29 (80.6)	0.97
Perineural invasion, n (%)	145 (92.4)	112 (92.6)	33 (91.7)	0.85
Adjuvant CT, n (%)	131 (80.3)	103 (83.7)	28 (75.6)	0.41

SD: standard deviation, IQR: interquartile range.

**Table 2:** Recurrence pattern.

	ER Group (n = 58)	NER Group (n = 80)	*p*-Value
Liver	8 (13.8)	18 (22.5)	*p* > 0.05
Lung	5 (8.6)	11 (13.8)	*p* > 0.05
Peritoneum	3 (5.2)	7 (8.8)	*p* > 0.05
Local	3 (5.2)	7 (8.8)	*p* > 0.05
More than 2 different region metastasis	39 (67.2)	37 (46.3)	*p* = 0.014

ER: early recurrence, NER: non-early recurrence.

**Table 3:** Preoperative risk factors associated with early recurrence.

Preoperative Risk Factors	Univariate OR (95%) *p* Value		Multivariate OR (95%) *p* Value	
Age (>70 vs. <70 years	2.02 (0.86–4.72)	0.103		
Gender (male vs. female)	1.63 (0.84–3.15)	0.146		
Charlson Comorbidity Index (>4 vs. <4)	1.85 (0.87–3.93)	0.108		
Tumor location (head/neck vs. body/tail)	2.69 (1.27–5.70)	0.010	2.55 (0.90–7.23)	0.076
ECOG (0 vs. 1–2)	1.86 (0.95–3.63)	0.068	3.31 (1.28–8.55)	0.013
Preoperative CA 19.9 (<208 U/mL vs. >208 U/mL)	4.21 (1.66–10.71)	0.002	3.18 (1.18–8.57)	0.022

OR: odds ratio.

**Table 4:** Postoperative risk factors associated with early recurrence.

Postoperative Risk Factors	Univariate OR (95%) *p*-Value		Multivariate OR (95%) *p*-Value	
Residual tumor (R0 vs. R1)	2.43 (1.2–4.7)	0.008	2.13 (0.85–5.35)	0.107
LVI (yes vs. no)	4.9 (1.61–14.84)	0.005		
PNI (yes vs. no)	0.14 (0.01–1.17)	0.070		
Tumor differentiation (well vs. modified/poor)	2.08 (1.05–4.1)	0.035	2.11 (0.84–5.28)	0.109
Tumor location (head vs. body/tail)	2.69 (1.27–5.70)	0.010	3.23 (1.08–9.64)	0.035
Tumor size (pathologically, <3.25 cm vs. >3.25 cm)	3.84 (1.77–8.31)	0.001	3.32 (1.21–9.04)	0.019
LNR (<0.13 vs. >0.13)	2.82 (1.33–5.99)	0.007	3.49 (1.34–9.08)	0.010
Adjuvant chemotherapy (yes vs. no)	5.74 (2.47–13.31)	<0.001	6.63 (1.81–24.72)	0.004

## Data Availability

The Authors agree to make data and materials supporting the results or analyses presented in their paper available upon reasonable request.

## Supplementary Materials

The following supporting information can be downloaded at: https://www.mdpi.com/article/10.3390/medicina61101799/s1, Table S1: Sensitivity analyses of early recurrence definitions at 3, 6, and 12 months; Table S2: Correlation matrix of postoperative variables, Table S3: ROC analysis of key variables.
