# Supplementary material for: Impact of Tumor Localization on Early Recurrence After Curative Resection in Pancreatic Ductal Adenocarcinoma
Source: Medicina (Kaunas). 2025 Oct 6;61(10):1799. doi: 10.3390/medicina61101799 (PMC12566036; doi:10.3390/medicina61101799)
Supplement: Supplementary file 1 [file medicina-61-01799-s001.zip › medicina-3786008-supplementary.pdf]

Supplementary Material

Supplementary Table S1. Sensitivity analyses of early recurrence (ER) definitions at 3, 6, and 12 months

Variable	ER ≤ 3 months (OR, 95% CI; p)	ER ≤ 6 months (OR, 95% CI; p)	ER ≤ 12 months (OR, 95% CI; p)
Tumour localisation (Body/Tail vs Head/Neck)	5.41(1.26-23.1, p=0.023)	3.23 (1.08-9.64, p=.035)	2.54 (.92-7.02), p=.07)
Tumour size > 3.25 cm	3.08(.76-12.3,p=.11)	3.32(1.21-9.04,p=.019)	2.24(.96-5.26,p=.06)
LNR > 0.13	1.98(.46-8.58,p=.35)	3.49(1.34-9.08,p=.01)	6.04(2.46-14.7,p=.00)
Adjuvant CT	19.4(4.4-85.3, p=.00)	5.74(2.47-13.3,p=.00)	2.35(.72-7.67,p=.15)

Sensitivity analyses demonstrated that tumour localisation was a significant predictor of recurrence when defined at 3 and 6 months, but not at 12 months.

Supplementary Table S2. Correlation matrix of postoperative variables

Variable	Tumour size	LNR	Adjuvant therapy	Localisation
Tumour size	1.000	.103	-.109	.106
LNR	.103	1.000	.298	.255
Adjuvant therapy	-.109	.298	1.000	.078
Localisation	.106	.255	.078	1.000

*Values are Pearson correlation coefficients (r). *p < 0.05, **p < 0.01.*

Supplementary Table S3. ROC analysis of key variables

Variable	Cut-off	AUC	95% CI	p-value
Tumour size	3.25 cm	0.657	0.529–0.773	0.017
CA19-9	208 U/mL	0.651	0.529–0.773	0.022
LNR	0.13	0.690	0.571–0.809	0.004

ROC analysis yielded modest discriminatory ability. All 95% CIs excluded 0.5, confirming statistically significant discrimination.
